# The role of diaphragmatic ultrasound as a predictor of successful extubation from mechanical ventilation in respiratory intensive care unit

**DOI:** 10.1186/s43168-021-00095-6

**Published:** 2021-11-16

**Authors:** Randa Salah Eldin Mohamed, Abeer Salah Eldin Mahmoud, Waleed Fouad Fathalah, Mohamed Farouk Mohamed, Ahmed Aelgharib Ahmed

**Affiliations:** 1 Department of Pulmonology, Beni Suef University Faculty of Medicine, Beni Suef, Egypt; 2 Department of Endemic Medicine and Hepatology, Cairo University Kasr Alainy Faculty of Medicine, Cairo, Egypt; 3 National Institute of Chest Hospital, 2 street Talat harb, Giza, Egypt

**Keywords:** Diaphragmatic thickening, Extubation, Diaphragmatic ultrasound, Thickening fraction, Excursion

## Abstract

**Background:**

The diaphragm muscle whose dysfunction may be very common in patients undergoing mechanical ventilation (Ferrari G, De Filippi G, Elia F, Panero F, Volpicelli G, Aprà F. Crit Ultrasound J 6:8, 2014). Aim: To evaluate real-time ultrasound in the evaluation of diaphragmatic thickening, thickening fraction, and/or excursion to predict extubation outcomes. We aimed to compare these parameters with other traditional weaning measures is a fundamental.

**Results:**

Out of 80 included patients, 20 (25%) have failed extubation. Diaphragmatic thickening (DT), thickening fraction (DTF), and/or excursion (DE) were significantly higher in the successful group compared to those who failed extubation (*p* < 0.05). Cutoff values of diaphragmatic measures associated with successful extubation (during tidal breathing) were ≥ 17 mm for DE; ≥ 2.1 cm for DT inspiration; ≥ 15.5 mm for DT expiration, functional residual capacity (FRC); and ≥ 32.82% for DTF %, giving 68%, 95%, 62%, and 90% sensitivity, respectively, and 65%, 100%, 100%, and 75% specificity, respectively. Cutoff values of diaphragmatic parameters associated with successful extubation (during deep breathing) were > 28.5 mm DT Insp, total lung capacity (TLC); >22.5mm DT Exp (RV); >37 DTF %; and > 31 mm DE, giving 100%, 73%, 97%, and 75% sensitivity and 65%, 75%, 100%, and 55% specificity, respectively. Rapid shallow breathing index (RSBI) had 47% sensitivity but 90% specificity.

**Conclusion:**

Ultrasound evaluation of diaphragmatic parameters could be a good predictor of weaning in patients who passed the T-tube.

## Background

The diaphragm is an important respiratory muscle, and dysfunction is very common in patients receiving mechanical ventilation. Diaphragm fatigue occurs even in patients who successfully pass the spontaneous breathing test (SBT) [1]. Interrupting ventilation too early can lead to increased cardiovascular and respiratory pressure (CO2) retention and hypoxemia with up to 25% of patients requiring reinstitution of ventilator support. Unnecessary delays in liberation from mechanical ventilation also can be deleterious. Complications such as ventilator-associated pneumonia and ventilator-induced diaphragm atrophy can be seen with short periods of mechanical ventilation, thereby prolonging mechanical ventilation [2]. As SBT monitoring is insensitive to detect early signs of load-capacity imbalance [3], the evaluation of the diaphragmatic thickening fraction (DTF) may be also helpful to assess diaphragmatic function and its contribution to respiratory workload [1]. Ultrasound can be used to detect the deflection of the diaphragm, which helps to identify patients with diaphragm dysfunction [4].

## Methods

This prospective study was carried out on 40 patients who are mechanically ventilated due to pulmonary disease, 40 patients on mechanical ventilation due to non-pulmonary disease at respiratory ICU, and 40 chronic obstructive pulmonary disease (COPD) patients from an outpatient clinic serving as controls at Embaba Chest Hospital, Cairo, Egypt, during a period from January 2018 to November 2019. Written informed consent was obtained from all patients prior to enrollment according to approval at the local committee of Beni-suef University Hospital. Patients on mechanical ventilation due to pulmonary disease (pneumonia, COPD, bronchial asthma, bronchiectasis …. etc.) and non-pulmonary disease (pulmonary edema, myocardial infarction, etc.) were included in this study. Patients with pneumothorax, pleural effusion, neuromuscular diseases, and suspicious diaphragmatic paralysis (raised copula in chest X-ray); patients with pleurodesis; and patients who presented with stridor (due to upper airway involvement due to mechanical ventilation in last 6 months) were excluded from this study.

### Study design

Patients were assessed by the following: Acute Physiology and Chronic Health Evaluation II (APACHE II) score, Charlson comorbidity index (CCI), and diaphragm ultrasound. M-mode ultrasound was used to assess diaphragmatic excursion, and movement B-mode ultrasound was used to assess diaphragmatic thickness. Once patients were stable and both ventilator and biochemical parameters were accepted for weaning, T-tube was attempted for 2 h. Patients who passed the SBT on T-tube were included in data analysis and followed up for 48h after extubation where they received oxygen through Venturi mask or nasal oxygen and followed up for 48 h after extubation. Successful extubation was defined as maintenance of spontaneous breathing for > 48 h following extubation. Extubation failure was defined as the inability to maintain spontaneous breathing for at least 48 h, without any ventilatory support. All patients were studied with the head of the bed elevated between 20 and 40°. Diaphragmatic thickness (DT) was measured using a 7–10-MHz linear ultrasound probe set to B-mode. The right hemidiaphragm was imaged at the zone of apposition of the diaphragm and rib cage in the midaxillary line between the 8th and 10th intercostal spaces. The DT was measured at end expiration and end inspiration. The percent change in DT between end expiration and end inspiration (DTF %) was calculated as (DT end inspiration − DT end expiration/DT end expiration) × 100 [5].

### Diaphragmatic excursion (DE)

**Fig. 1:**
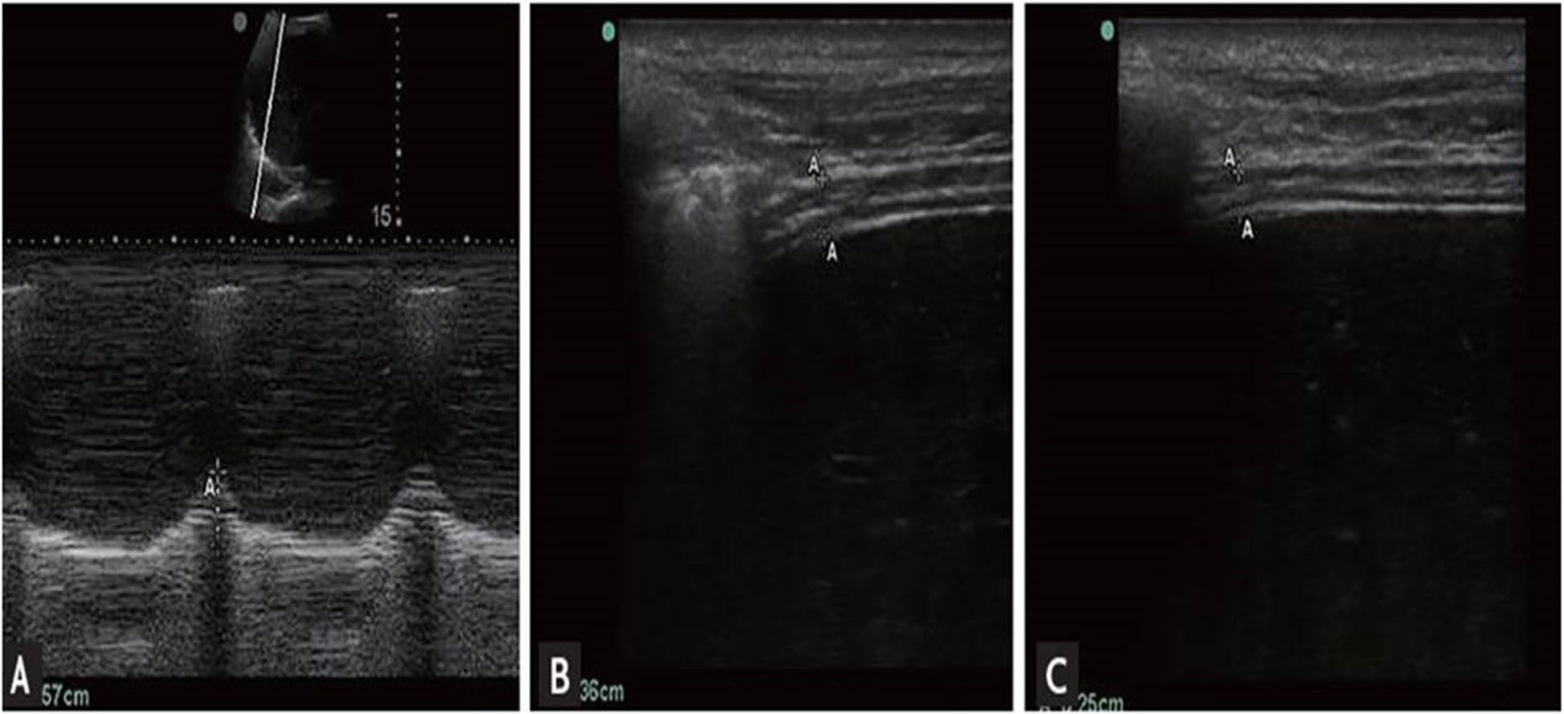
M-mode of diaphragm excursion (**A**) and B-mode of diaphragm thickness (**B**, inspiration; **C**, expiration)

The convex probe is placed in the right subcostal region parallel to the intercostal space to measure the range of the diaphragmatic movement using the M-mode method with the cursor crossing the diaphragm to assess the highest and lowest points as an indicator for the diaphragmatic mobility range [6, 7]. The maneuver was repeated at least three times and the average measurement is taken. Measurement of diaphragmatic thickness and excursion was recorded during tidal breathing and deep breathing (Fig. 1).

### Criteria of weaning

The criteria of weaning are 1- positive end-expiratory pressure (PEEP) ≤ 5 cm H2O 2- Fraction of inspired oxygen (FiO2) < 0.5 3- Respiratory rate (RR) < 30 breaths/min 4- rapid shallow breathing index < 105, and PaO2/FiO2 > 200.

### Criteria for failure

The criteria for failure are change in mental status, onset of discomfort, diaphoresis, respiratory rate > 35 breaths/min, and hemodynamic instability (heart rate > 140, systolic blood pressure >180) [8]. Patients were divided into two groups: group A included 40 patients on mechanical ventilation due to pulmonary diseases to compare parameters of weaning to diaphragmatic thickness and excursion during tidal breathing and deep breathing. Group B includes 40 patients on mechanical ventilation due to non-pulmonary diseases to compare parameters of weaning to diaphragmatic thickness and excursion during tidal breathing and deep breathing.

### Statistics

The collected data was revised, coded, tabulated, and introduced to a PC using the Statistical Package for Social Science (SPSS 17). Data was presented and suitable analysis was done according to the type of data obtained for each parameter. The distributions of quantitative variables were tested for normality. Quantitative data were described using mean and standard deviation for normally distributed data while abnormally distributed data was expressed using the median. For normally distributed data, comparisons between both groups were done using an independent *t*-test, while abnormally distributed data was assessed using the Mann-Whitney test. A receiver operator characteristic curve (ROC curve) was used to find out the best cutoff value and the validity of a certain variable. Agreement of the different predictive values of the outcome was used and was expressed in sensitivity, specificity, positive predictive value, and negative predictive value.

## Results

**Fig. 2:**
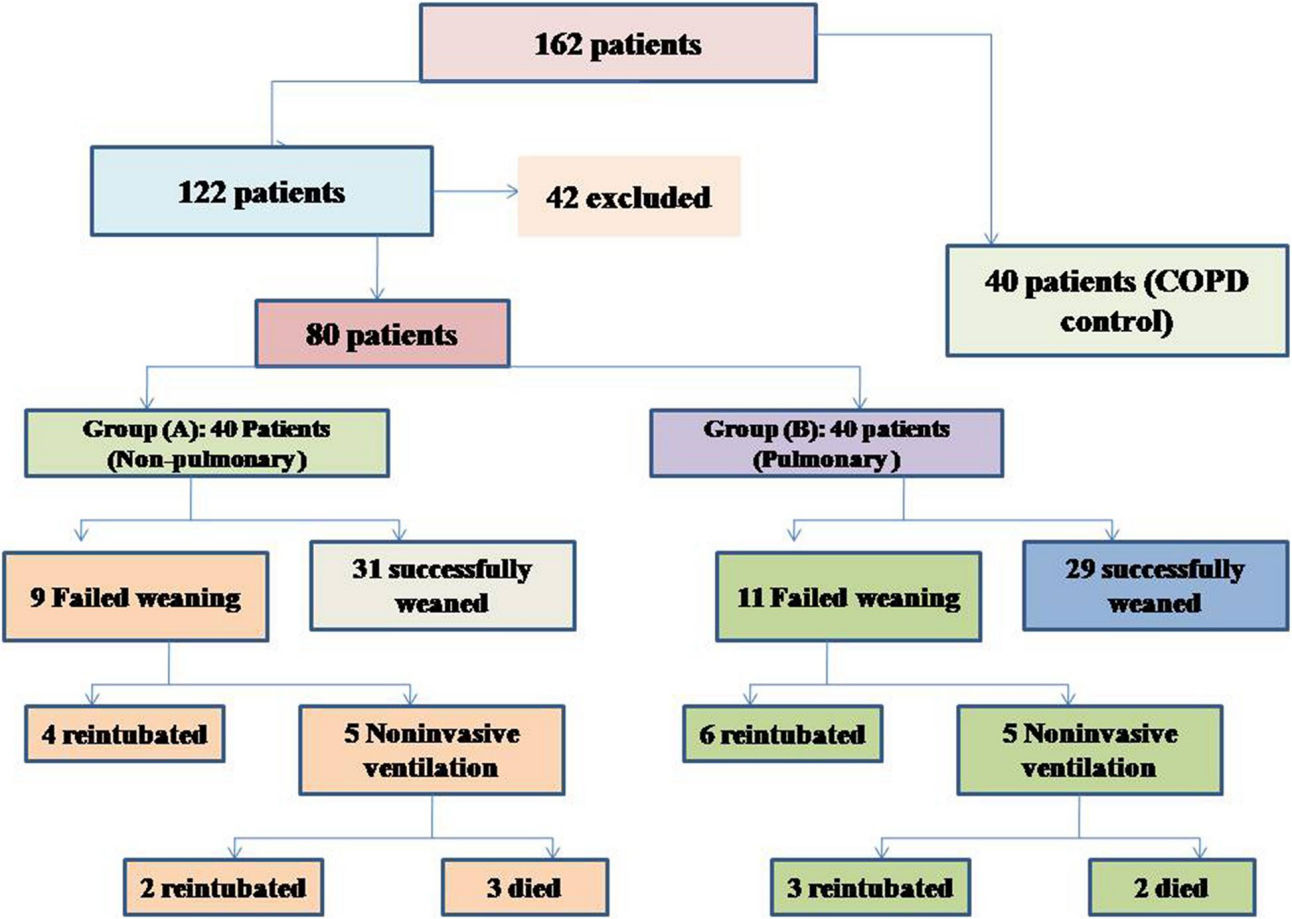
Flow chart showing criteria of patients’ selection and follow-up through the study

**Fig. 3:**
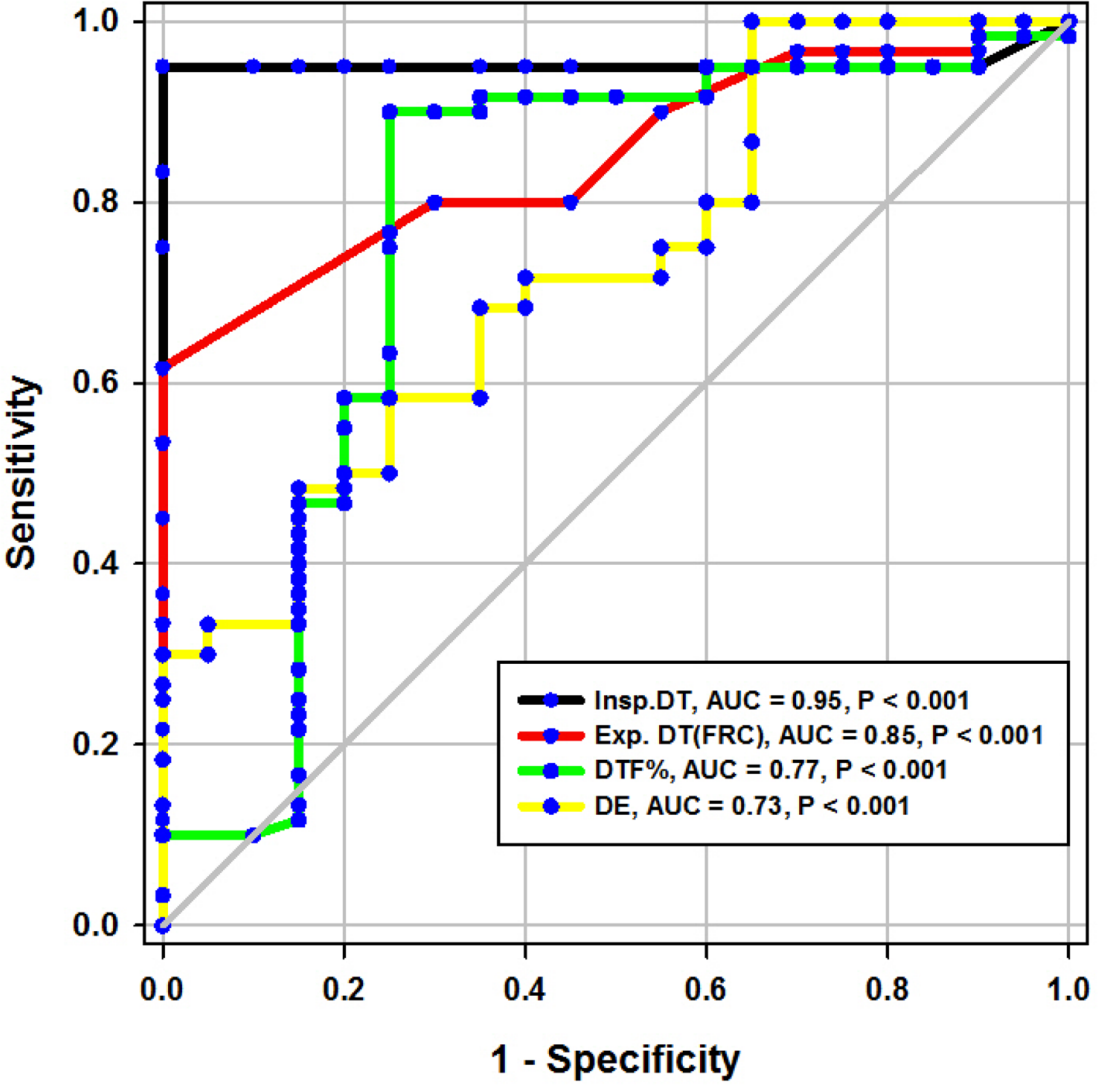
ROC curve of diaphragmatic parameters during tidal breathing

**Fig. 4:**
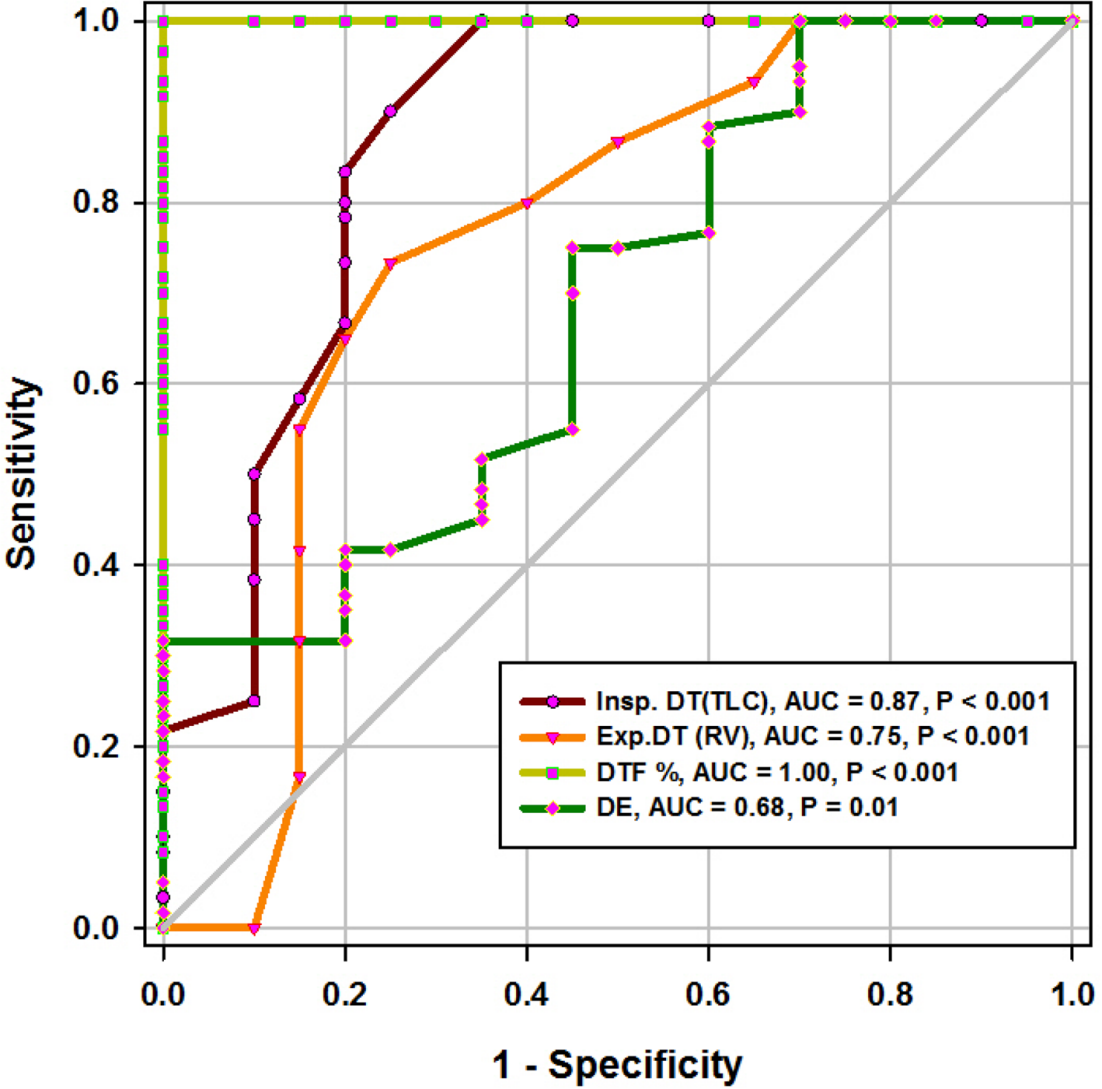
ROC curve of diaphragmatic parameters during deep breathing

**Fig. 5:**
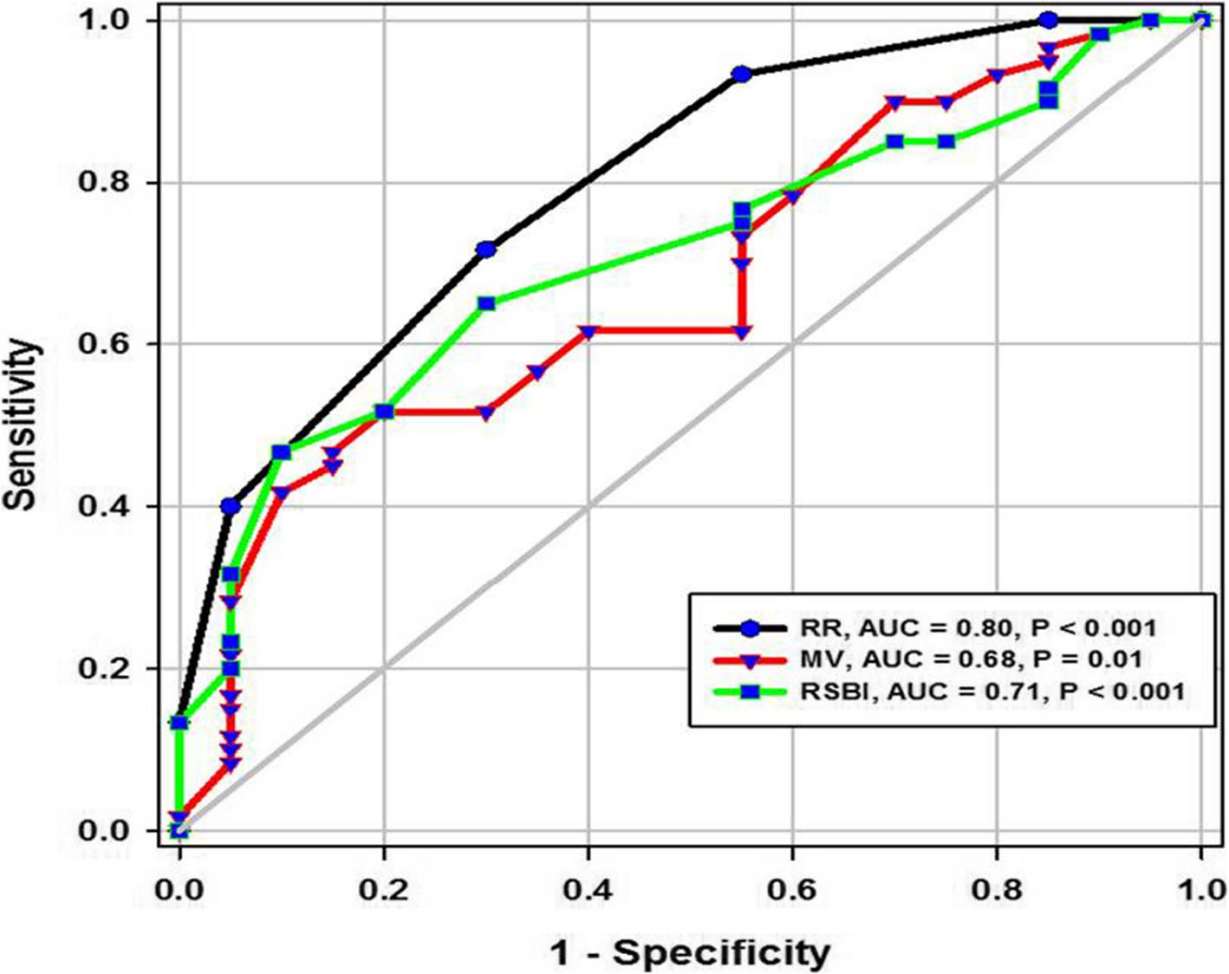
ROC curve of RR, MV, and RSBI in the prediction of successful weaning

During the study period (Fig. 2), we evaluated 162 patients ready for weaning. Forty chronic obstructive pulmonary disease (COPD) patients (stable) served as a control group. Forty-two patients were excluded, 10 of which had pleural effusion, 4 patients had pneumothorax, 10 patients had diaphragmatic paralysis, and 18 patients were non-cooperative. Eighty patients (on T-tube) undergoing SBT were divided into two groups: group A included 40 patients (non-pulmonary-related cause) and had their diagnosis as follows: 24 (60%) had congestive heart failure, 4 (10%) had diabetes mellitus, 4 (10%) had sepsis other than pneumonia, 2 (5%) had epilepsy, 2 (5%) had embolic hemiplegia, and 4 (10%) had chronic renal failure. Out of group A patients, 9 patients (11.25%) had failed weaning of which 4 patients needed reintubation and 5 patients needed non-invasive positive ventilation of which 2 patients were reintubated and 3 patients died. Group B included 40 patients (pulmonary-related cause) and had their diagnosis as follows: 21 (53%) had COPD, 8 (20%) had asthma, 5 (13%) had bronchiectasis, 5 (13%) had pneumonia, and 1 (3%) had viral influenza H1N1. Out of group B patients, 11 patients (13.75%) had failed weaning, of which 6 patients needed reintubation and 5 patients needed non-invasive positive ventilation of which 3 patients were reintubated and 2 patients died. Regarding ultrasound diaphragmatic parameters (during tidal breathing) (Table 1), DT Insp mm, DT Expt (FRC) mm, DTF %, and DE in centimeters were significantly higher [24 mm (23.25–26) vs.18 mm (17–19.15), *p* < 0.001; 17 mm (15–18) vs.14 mm (12.3–15), *p* < 0.001; 44.41% (35.07–67.12) vs. 30.38% (23.34–38.07), *p* < 0.001; 1.95 cm (1.53–2.75) vs. 1.66 cm (1.09–1.94), *p* <0.003]. Regarding ultrasound diaphragmatic parameters during deep breathing (Table 1), DT Insp (TLC) mm, DT Exp (RV) mm, DTF %, and DE in cm were significantly higher [36.5 mm (33–39.75) vs. 26 mm (23.25–29.75), *p* < 0.001; 25 mm (22–27) vs. 20.5 mm (18–22.75), *p* < 0.001; 50% (43.05–58.2) vs. 25% (23.8–26.99), *p* < 0.001; 3.6 cm (3–5.4) vs. 2.95 cm (1.73–4.05), *p* < 0. 0.01] respectively in the successfully extubated group compared to the failed one (Table 1). AUC was used to assess the accuracy of diaphragmatic parameters in predicting failed extubation (during tidal breathing) (Table 2) (Fig. 3). A cutoff value of DT Exp (FRC) > 15.5mm was associated with successful extubation with 62% sensitivity and 100% specificity, a cutoff value of DTF % > 32.82 was associated with successful extubation with 90% sensitivity and 75% specificity, a cutoff value of DE > 1.7 cm was associated with successful extubation with 68% sensitivity and 65% specificity, and the optimum cutoff value of DT Insp > 21 mm was associated with successful extubation with 95% sensitivity and 100% specificity (Table 2) (Fig. 3). A cutoff value (during deep breathing) of DT Exp (RV) > 22.5 was associated with successful extubation with 73% sensitivity and 75% specificity, a cutoff value of DE > 3.1 was associated with successful extubation with 75% sensitivity and 55% specificity, a cutoff value of DT Insp (TLC) > 28.5mm was associated with successful extubation with 100% sensitivity and 65% specificity, and the optimum cutoff value of > 37 DTF % was associated with successful extubation with 97% sensitivity and 100% specificity but AUC 100% (Table 2) (Fig. 4). Among the traditional weaning parameter (RSBI, minute ventilation, RR, and PaO2/FiO2), PaO2/FiO2 was significantly more in the successful extubation group than the failed one [206 (197.3–211.8) vs. 190 (185–199.8), *p* < 0.0.001] (Table 1) (Fig. 5). All DT parameters were significantly higher in the COPD group than in failed weaning in the pulmonary group (B) (Table 3).

**Table 1:** Comparison between patients with successful and failed weaning as regards traditional weaning indices and new diaphragmatic parameters

Variables	Successful weaning (*n* = 60)		Failed weaning (*n* = 20)		*p*
Traditional weaning					
TV (mL/kg)	435	(420–458.8)	450	(420–460)	0.4^$$^
PaO_2_/FIO_2_	206	(197.3–211.8)	190	(185–199.8)	< 0.001^$$^
RR	16	(15–17)	18	(17–19)	< 0.001^$$^
MV (L/m)	6.83	0.71	7.31	0.77	0.01^$^
RSBI	58	(52–63)	46	(41–51)	0.005^$$^
During tidal breathing					
DT Insp mm	24	(23.25–26)	18	(17–19.15)	< 0.001^$$^
DT Expt(FRC) mm	17	(15–18)	14	(12.3–15)	< 0.001^$$^
DTF%	44.41	(35.07–67.12)	30.38	(23.34–38.07)	< 0.001^$$^
DE in cm	1.95	(1.53–2.75)	1.66	(1.09–1.94)	0.003^$$^
During deep breathing					
DT Insp (TLC) mm	36.5	(33–39.75)	26	(23.25–29.75)	< 0.001^$$^
DT Exp (RV) mm	25	(22–27)	20.5	(18–22.75)	< 0.001^$$^
DTF %	50	(43.05–58.2)	25	(23.8–26.99)	< 0.001^$$^
DE in cm	3.6	(3–5.4)	2.95	(1.73–4.05)	0.01^$$^

Numerical data is represented as mean and SD or median and IQR. *N* number, *SD* standard deviation, *IQR* interquartile range. ^$^Independent *t*-test, ^$$^Mann-Whitney test, *p* is considered significant if < 0.05

**Table 2:** Diagnostic accuracy of a new weaning index (diaphragmatic index) with tidal breathing, during deep breathing and traditional weaning

Variables	Cutoff	Sensitivity	Specificity	PPV	NPV	AUC	*p*
Traditional weaning							
PaO_2_/FIO_2_	>202.5	61%	95%	73%	92%	84%	< 0.001
RR	<16.5	72%	70%	34%	92%	80%	< 0.001
MV(L/m)	<8.7	42%	90%	47%	87%	68%	0.01
RSBI	<35.5	47%	90%	51%	188%	71%	< 0.001
During tidal breathing							
DT Insp	> 21	95%	100%	100%	99%	95%	< 0.001
DT Exp (FRC)	>15.5	62%	100%	100%	92%	85%	< 0.001
DTF%	>32.82	90%	75%	44%	97%	77%	< 0.001
DE	>1.7	68%	65%	30%	90%	73%	< 0.001
During deep breathing							
DT Insp (TLC)	>28.5	100%	65%	39%	100%	87%	< 0.001
DT Exp (RV)	>22.5	73%	75%	39%	93%	75%	< 0.001
DTF %	>37	97%	100%	100%	99%	100%	< 0.001
DE	>3.1	75%	55%	27%	91%	68%	0.01

*PPV* positive predictive value, *NPV* negative predictive value, *AUC* area under a curve, *p* is considered significant if < 0.05

**Table 3:** Comparison between COPD patient (control) and patient with failed weaning in the pulmonary group (B)

Variables	Failed weaning (*n* = 11)		COPD (*n* = 40)		*p*^$^
During tidal breathing					
DT Insp mm	18.12	16.5	36.1	69	< 0.001
DT Expt (FRC) mm	13.79	16.4	31.8	88	< 0.001
DTF%	39.20	22.10	39.50	23	0.04
DE in cm	1.52	0.55	2.36	56	< 0.001
During deep breathing					
DT Insp (TLC) mm	28.27	56.6	55.3	57	< 0.001
DT Exp (RV) mm	22.45	42.5	31.3	44	< 0.001
DTF %	25.33	2.43	71.00	2.780	< 0.001
DE in cm	2.95	1.24	3.74	1.35	0.04

Numerical data is represented as mean and *SD*, *N* number, *SD* standard deviation, ^$^independent *t*-test, *p* is considered significant if < 0.05

## Discussion

The diaphragm is the main respiratory muscle, which plays an important role in the respiratory movement, and its dysfunction predisposes to prolonged duration of mechanical ventilation and respiratory complications. Sonographic evaluation has recently started to become popular in the intensive care unit (ICU) for assessing diaphragmatic function [9]. In comparing the control COPD cases with others who suffered from MV with failed weaning experience, regarding US parameters during tidal breathing, both of inspiratory, expiratory DT, DE, and DTF % were significantly higher in the COPD group (control) than in the failed weaning group (B) (*p* < 0.001). Furthermore, during deep breathing techniques, all DT parameters were significantly higher in the COPD group than in the weaning failure group (*p* < 0.001). In our knowledge, this is the first study that compared pulmonary diseases and COPD as regards the diaphragmatic ultrasound parameter (Table 3).

### Diaphragmatic thickness during tidal breathing (Fig. 3)

In the present study, DT at end inspiration in the successful group was 24 mm (23.25–26), versus failed group 18 mm (17–19.15), *p* < 0.001, with a cutoff point > 21mm, 95% sensitivity, 100% specificity, 100% PPV, 99% NPV, and an AUC 95% (Tables 1 and 2). Similarly, Farghaly and Hasan [3] found DT at end inspiration in a successful group was 24 mm (22–28), versus failed group 18 mm (15–20), with a cutoff point ≥ 21 mm, 77.5% sensitivity, 86.6% specificity, and an AUC of 83.1%. In the present study, DT (FRC) at end expiration in a successful group was 17 mm (15–18), versus failed group 14 mm (12.3–15), *p* = 0.001, with a cutoff point >15.5%, 62% sensitivity, 100% specificity, 100% PPV, 92% NPV, and an AUC 85% (Tables 1 and 2) (Fig. 3). Similarly, Farghaly and Hasan found that DT at end expiration in a successful group was 16 mm (11.2–18.7), versus failed group 11 mm (10–15), with a cutoff point ≥ 10.5 mm, 80% sensitivity, 50% specificity, and an AUC 68.8% [3]. In the present study, DTF% in a successful group was 44.41% (35.07–67.12), versus failed group 30.38% (23.34–38.07), with a cutoff point > 32.82%, 90% sensitivity, 75% specificity, 44% PPV, 97% NPV, and an AUC 77% (Tables 1 and 2) (Fig. 3). This result is consistent with studies by Farghally and Hasan [3] and Dinino et al. [10] which demonstrated that DTFs with a cutoff point more than 34 and 30, respectively, were associated with weaning success and better ICU outcomes. In contrast with Umbrello et al. [4], who observed patients after major elective surgery and first weaning failure, they reported that a cutoff point of DTF more than 20% was associated with weaning success, and this may be explained by the absence of surgical patients in this study. In the present study, DE in a successful group is 1.9 cm (1.53–2.75), versus failed group 1.66 cm (1.09–1.94), *p* = 0.001, with a cutoff point > 1.7 cm, 68% sensitivity, 65% specificity, 30% PPV, 90% NPV, and an AUC 0.73 (Tables 1 and 2 ) (Fig. 3). This result is consistent with the studies done by Matamis et al. [9] and Palkar et al. [11] who confirmed that DE at a cutoff point of more than 1.65 cm and 1.64 cm, respectively, was associated with weaning success and better ICU outcomes. Also, Gursel et al. [12] reported that tidal diaphragmatic excursion using standard ultrasound devices (SD) is 1.76 ± 0.69 cm (0.58–3.30) and using pocket-sized ultrasound devices (PSDs) 1.62 ± 0.70 cm (0.50–3.00). In the present study, the AUC of the DT Insp (95) was more than that of DTF (77), while AUC of DT Exp (FRC) (85) was more than that of DTF (77). In contrast, Farghaly and Hasan stated that AUC of DT (83.1) at end inspiration was more than DT (68.8) at the end expiration and AUC of DT (68.8) at the end expiration was less than DTF (70. 8). Also, it was found that AUC of DT (61) at the end expiration was less than that of DTF (79) alone [3]. In the present study, the DE was less (68%) sensitive than that DT Insp (95%), and the specificity of DT Insp (100%) was more than that of DE (30%) (Table 2). Similarly, Farghaly and Hasan observed that diaphragm excursion should not be used in the assessment of diaphragmatic contractile activity, whereas diaphragm thickening is a good indicator of respiratory effort [3]. Also, Umbrello et al. observed that during pressure support ventilation, diaphragm thickening was more accurate than diaphragm excursion and suggested that the use of diaphragm excursion is of little help during PSV and should not be used in the assessment of diaphragmatic contractile activity [4]. In contrast, Hayat et al. [13] reported that diaphragmatic excursion is a good method for predicting the weaning outcome.

### Diaphragmatic thickness during deep breathing

In the current study, diaphragm thickness at TLC in a successful group was 36 mm (33–39.75), versus failed group 26 mm (23.25–29.75) with a cutoff point 28.5, 100% sensitivity, 65% specificity, 39% PPV, 100% NPV, and an AUC 0.87, while diaphragm thickness at RV in the successful group was 25 mm (22–27), versus failed group 20.5 mm (18–22.75) with a cutoff point 22.5 mm, 73% sensitivity, 75% specificity, 39% PPV, 93% NPV, and an AUC 0.75 (Tables 1 and 2) (Fig. 4). Similarly, Ferrari et al. stated that diaphragm thickness (DT) at TLC in a successful group was 38 mm (29–45), versus failed group 30 mm (20–40) [1], while DT at RV in a succeeded group was 25 mm (19–28), versus failed group 24 mm (17–30). Moreover, Gursel et al. found that the maximal inspiratory thickness was SD 47 ± 16mm (23–68) and PSDs 45 ± 12mm (24–91). In contrast, Pirompanich and Romsaiyut noted that DT at TLC in a succeeded group was 35 ± 13 and 38 mm (IQR 29–45), versus failed group 31 ± 13 mm and 30 mm (IQR 20–40) [12], while diaphragm thickness at RV in a successful group was 22 ± 09 mm and 25 mm (IQR 19–28), versus failed group 25 ± 11 mm and 24 mm (IQR 17–30).There were higher values about RV in the failed group more than the successful group, and these variables can be explained by different causes for mechanical ventilation as well as different ventilation periods and different ethnic groups which may affect the thickness of the diaphragm. In the present study, DTF in a successful group was 50% (43.05–58.20), versus failed group 25% (23.80–26.99), with a cutoff point of 37%, 97% sensitivity, 100% specificity, 97% PPV, 100% NPV, and an AUC 1 (Tables 1 and 2) (Fig. 4). These results are consistent with studies done by Ferrari et al. [1] which demonstrated that DTFs of more than 36% were associated with weaning success and better ICU outcomes. Our study found that DE in a successful group was 3.6 cm (3–5.4), versus failed group 2.95 cm (1.73–4.05), with a cutoff point DE 3.1 cm, 75% sensitivity, 55% specificity, 27% PPV, 91% NPV, and an AUC 0.68 (Tables 1 and 2) (Fig. 4). Similarly, Carrie et al. found that DE in the successful group was 4.1 ±2. 1cm, versus failed group 3 ± 1.8cm with a cutoff point DE 2.7cm [14]. Also, Gursel et al. found in their study DE (±SD) was 2.97 ± 1.18cm (1.33–5.40) and PSDs 2.67 ± 0.90cm (1.30–4.70) [12]. Moreover, Lerolle et al. reported that DE less than 2.5 cm was a predictor of weaning failure, in post-cardiac patients connected to mechanical ventilation [15]. In the present study, the DTF was more specific and sensitive with a higher AUC (100%, 97%, 1) than DE (55%, 75%, 0.91) (Table 2) (Fig. 4). This result is consistent with the studies by Samanta et al. [16] and Ferrari et al. [1] who reported that the DTF is more accurate than DE in the prediction of successful weaning. In the present study, DT Insp (TLC) is more sensitive and specific (100%, 65%) than DE (75%, 55%). The AUC of DT Insp (TLC) was more than that of DT Exp (RV) (0.87 and 0.75, respectively). The AUC of DTF was more than the AUC of DT Insp (TLC) (100 and 87, respectively) (Table 2) (Fig. 4). In contrast, Farghaly and Hasan observed that the AUC of DT at end inspiration was more than DT at end expiration (83.1 and 68. 8, respectively) [3]. Also, Di Nino et al. observed that the AUC for DT end expiration was less than that for DTF% alone (0.79 and 0.61, respectively) [10]. However, they determined DT, DTF, and DE during tidal breathing, while in the current study, DT, DTF, and DE were assessed during tidal and deep breathing. In the present study, the AUC of DTF during deep breathing was more than DT Insp during tidal breathing (100 and 95, respectively), while the AUC of DT Insp was more than DT Insp (TLC) (95 and 87, respectively) (Table 2). In the present study, the RSBI in the successful group was 58 (52–63) breath/min/L, versus failed group 46 (41–51) breath/min/L, *p* < 0.005, and a cutoff value for RSBI was 35.5 b/min with 47% sensitivity, 90% specificity, 51% PPV, 188% NPV, and the AUC of 71% in predicting extubation failure (Tables 1 and 2) (Fig. 5). Similarly, Farghaly and Hasan observed that the RSBI in a successful group was 51.5 (44–79), versus failed group 50 (40–65), *p* <0.005 [3]. Also, Pirompanich and Romsaiyut found that the average RSBI in a successful group was 54. 3 ± 22.8, versus failed group 47.7 ± 14.8, *p* < 0.012 [14]. In contrast, Ferrari et al. observed that the RSBI in a successful group was 70 (57–83), versus failed group 120 (110–148), *p* < 0.0001 [1]. This variation can be explained by different causes for mechanical ventilation as well as different ventilation periods, which may affect the outcome of the weaning process. During tidal breathing, the specificity of RSBI was less than DT at insp and DT Exp (FRC) at end expiration (90, 100, and 100). But the specificity of RSBI is more than DTF and DE (90, 75, and 65). But the AUC of RSBI is less than DT Insp, DT Exp (FRC), DTF, DE, TLC, RV, and DTF (71, 95, 85, 77, 73, 87, 75, and 100, respectively). The AUC of RSBI during forced expiration and inspiration is more than DE (71 and 68, respectively) (Table 2) (Figs. 3, 4, and 5). Similarly, DiNino et al. reported that the diaphragmatic thickness and diaphragmatic thickness fraction are more accurate than RSBI, for predicting successful weaning [10]. Also, Pirompanich and Romsaiyut observed that integration of DTF (right) (AUC 95%) and RSBI (AUC 70%) are more accurate than RSBI (AUC 70%), for foretelling of successful extubation [17]. Similarly, Farghaly and Hasan reported that the diaphragm thickness, DTF, and DE during tidal breathing are more accurate than RSBI [3]. They recommended to consider the use of these parameters with RSBI to improve weaning outcome. In addition, Hayat et al. reported that the DE during tidal breathing is more accurate than RSBI, but they did not use DT and DTF in the comparison [13]. Ramakrishnan and Siddiqui reported that the diaphragmatic excursion is probably better in predicting extubation success than RSBI [18].

### Fate of the studied patients

In the present study, as regards group A, the number of patients with successful weaning was 31 (77.5%) versus 9 (22.5%) of weaning failure, while in group B, the number of patients with successful weaning was 29 (72.5%) versus 11 (27.5%) of weaning failure. This is consistent with Esteban et al. [8], 27%. This is in contrast with Ferrari et al. [1] who reported a 63% failure rate. This variation can be explained by different causes for mechanical ventilation as well as different ventilation periods before starting the weaning process, which may affect the outcome of the weaning process.

### Study limitations

The measurements of the diaphragm were not supplemented with direct measurements (such as the maximal expiratory pressure, maximal inspiratory pressure, and transdiaphragmatic pressure). This study was done in the respiratory care unit, and there were no surgical treated patients. While the (reference) thickness of the diaphragm in many diseases, e.g., COPD, pneumonia, and DM, is still unknown, the golden standard of measuring the diaphragmatic strength is phrenic nerve stimulation, and comparing it with sonographic findings was not done in this study. This study did not target a certain chest disease in its assessment of the diaphragm. The right hemidiaphragm was used in the diaphragmatic assessment being easier in imaging than the left hemidiaphragm which is often impeded by intestinal and gastric gas.

## Conclusions

Ultrasound of the diaphragm is a simple, easy, non-invasive, and inexpensive method useful to evaluate the thickness of the diaphragm in the zone of apposition. Assessment of DT, DTF by diaphragm ultrasound in B-mode, and DE in M-mode represents a new weaning index with highly accurate results in comparison to the other traditional indices as RSBI, so they can be used as predictive parameters to assess the weaning process outcome.

## Quick look

Ultrasound of the diaphragm is a simple, easy, non-invasive, and inexpensive method useful to evaluate the diaphragmatic muscle. Parameters like diaphragmatic thickness and diaphragmatic excursion can be recorded by real-time ultrasound and could have many clinical reflections. The diaphragmatic thickness fraction during deep breathing could be a good foreteller of weaning from mechanical ventilation.

### What this paper contributes to our knowledge

Assessment of diaphragmatic thickness, by diaphragmatic ultrasound in B-mode and diaphragmatic excursion in M-mode, can be used as predictive parameters to assess the weaning process outcome in patients on mechanical ventilation.

## Data Availability

The datasets generated during and/or analyzed during the current study are available from the corresponding author on reasonable request.

## Abbreviations

APACH II: Acute Physiology and Chronic Health Evaluation II
B-mode: Brightness mode
CCI: Charlson comorbidity index
COPD: Chronic obstructive lung disease
CMV: Control mechanical ventilation
CT: Computerized tomography
DB: Decibel
DE: Diaphragmatic excursion
DM: Diabetes mellitus
DT: Diaphragmatic thickness
DT Exp: Diaphragmatic thickness at the end expiration
DTF: Diaphragmatic thickness fraction
DT Insp: Diaphragmatic thickness at end inspiration
DUS: Diaphragmatic ultrasound
FRC: Functional residual capacity
FTUS: Focus thoracic ultrasound
HTN: Hypertension
ICU: Intensive care unit
IHD: Ischemic heart disease
IQR: Interquartile range
IVC: Inferior vena cava
LUS: Lung ultrasound
MHz: MegaHertz
M-mode: Motion mode
MRI: Magnetic resonance imaging
MV: Mechanical ventilation
NIV: Non-invasive ventilation
NPV: Negative predictive value
PMV: Prolonged mechanical ventilation
PPV: Positive predictive value
PSDs: Pocket-sized ultrasound devices
PSV: Pressure support ventilation
PTD: Transdiaphragmatic pressure
RSBI: Rapid shallow breathing index
RV: Residual volume
SBT: Spontaneous breathing
SUD: Standard ultrasound devices
SD: Standard deviation
SPL: Spatial pulse length
TDI FRC: Diaphragm thickness at functional residual capacity
TLC: Total lung capacity
TUS: Thoracic ultrasound
US: Ultrasound
VIDD: Ventilator-induced diaphragmatic dysfunction
VT: Tidal volume
2D: Two-dimensional
3D: Three-dimensional
